# Phenylarsine Oxide Can Induce the Arsenite-Resistance Mutant PML Protein Solubility Changes

**DOI:** 10.3390/ijms18020247

**Published:** 2017-01-25

**Authors:** Yu Han Jiang, Ye Jia Chen, Chao Wang, Yong Fei Lan, Chang Yang, Qian Qian Wang, Liaqat Hussain, Yasen Maimaitiying, Khairul Islam, Hua Naranmandura

**Affiliations:** 1 Department of Toxicology, School of Medicine and Public health, Zhejiang University, Hangzhou 310058, China; 21434054@zju.edu.cn (Y.H.J.); cyjdota@163.com (Y.J.C.); 11618280@zju.edu.cn (C.W.); 21419019@zju.edu.cn (Y.F.L.); 11518283@zju.edu.cn (C.Y.); 11319039@zju.edu.cn (Q.Q.W.); liaqathussain@zju.edu.cn (L.H.); yasinjan@zju.edu.cn (Y.M.); drkhairul16@gmail.com (K.I.); 2 Department of Marine Science, Ocean College, Zhejiang University, Hangzhou 310058, China; 3 Department of Pharmacology, School of Medicine, Zhejiang University, Hangzhou 310058, China; 4 College of Pharmaceutical Sciences, Zhejiang University, Hangzhou 310058, China

**Keywords:** arsenite, acute promyelocytic leukemia, arsenic trioxide, monomethylarsonous acid, dimethylarsinous acid, phenylarsine oxide, trivalent arsenicals

## Abstract

Arsenic trioxide (As_2_O_3_) has recently become one of the most effective drugs for treatment of patient with acute promyelocytic leukemia (APL), and its molecular mechanism has also been largely investigated. However, it has been reported that As_2_O_3_ resistant patients are frequently found in relapsed APL after consolidation therapy, which is due to the point mutations in B-box type 2 motifs of *promyelocytic leukemia* (*PML*) gene. In the present study, we for the first time establish whether organic arsenic species phenylarsine oxide (PAO) could induce the mutant PML-IV (A216V) protein solubility changes and degradation. Here, three different PML protein variants (i.e., PML-IV, PML-V and mutant PML-A216V) were overexpressed in HEK293T cells and then exposed to PAO in time- and dose-dependent manners. Interestingly, PAO is found to have potential effect on induction of mutant PML-IV (A216V) protein solubility changes and degradation, but no appreciable effects were found following exposure to high concentrations of iAs^III^, dimethylarsinous acid (DMA^III^) and adriamycin (doxorubicin), even though they cause cell death. Our current data strongly indicate that PAO has good effects on the mutant PML protein solubility changes, and it may be helpful for improving the therapeutic strategies for arsenic-resistant APL treatments in the near future.

## 1. Introduction

Arsenic trioxide (As_2_O_3_) has been widely used in traditional medicine for treatment of various diseases for more than two thousand years [1]. Recently, it has been established as one of the most effective drugs for treatment of patient with acute promyelocytic leukemia (APL), as well as applied to other types of solid malignant tumors and diseases as clinical treatment [2,3].

APL is a subtype of acute myeloid leukemia (AML) with distinctive biological and clinical features that has recently been found to be highly curable by all-trans retinoic acid (ATRA) and As_2_O_3_ treatment [4,5]. Additionally, APL is characterized by the reciprocal chromosomal translocation t(15;17)(q22;q21), resulting in the fusion of *promyelocytic leukemia* (*PML*) gene to the *retinoic acid receptor* α (*RAR*α) gene, and, finally, the expression of PML-RARα fusion protein to block cell differentiation. Fortunately, this specific genetic lesion determines the unique response to treatment with As_2_O_3_ or all-trans retinoic acid (ATRA), especially As_2_O_3_ has dual effects on inducing partial differentiation and apoptosis of APL cells, differently from ATRA [6,7,8].

Indeed, As_2_O_3_-induced PML-RARα degradation is the basis of its clinical efficacy in APL [9,10]. Additionally, it has been found that As_2_O_3_ directly targeted PML moiety of PML-RARα fusion protein and causes its fusion protein degradation resulting in clinical remission [9,10]. In fact, PML protein contains cysteine-rich zinc binding domains, a RING (R), a B-box type 1 (B1) and a B-box type 2 (B2) motifs, followed by a Coiled-Coil (CC) region, namely (RBCC) [11]. It has already been reported that rapid degradation of PML-RARα fusion protein was considered to be associated with direct interaction of As_2_O_3_ by targeting cysteine residues in RBCC domains of PML, and consequent induction of PML-RARα conformational changes, oligomerization and then modified by SUMOylation as well as ubiquitination, finally resulting in clinical remission by PML-RARα fusion protein degradation [12,13,14]. Although As_2_O_3_ has significant clinical effectiveness in the treatment of APL, it has been reported that arsenic resistant patients are commonly found in relapsed APL after consolidation therapy, and their outcomes are extremely poor [15]. Several studies have shown that point mutations in B-box2 of PML or PML-RARα fusion genes are frequently observed [16], and all mutant PML proteins have exhibited strong resistance to As_2_O_3_ treatment in vitro [17,18].

To date, more than nine mutations have been found in PML, and most mutations were identified in B-box 2 motif, such as A216V, L218P, C212A, C213A, S214L, A216T, L217F, etc. [19]. However, it still remains unknown why As_2_O_3_ loses its ability to induce degradation of these mutants PML protein. On the other hand, without PML mutations, APL patients have exhibited no resistance to arsenic trioxide [17], indicating that the binding of As_2_O_3_ in the RBCC domain appears to be critical for the effect of arsenic on PML-RARα degradation; more specifically, Cys77/80 and Cys88/91 in the RING domain as well as Cys212 and Cys213 in the B2 domain are found to be arsenic targeting sites [12].

In humans, at least seven PML isoforms (PML-I to PML-VII) have been identified, and all isoforms are generated by alternative splicing of the primary *PML* gene [11,20,21]. Additionally, these isoforms share a common N-terminal region, which includes the RBCC domain, but differ in their C-terminal regions due to alternative splicing of exons and account for isoform-specific functions [22]. Interestingly, PML isoforms I–VI are localized in nucleus, and PML nuclear bodies (PML-NBs) are currently suggested to be formed by these six isoforms, while the remaining isoform, PML-VII, is localized in cytoplasm [23]. Moreover, PML-IV and -V are commonly used to establish the effect of As_2_O_3_ on PML protein solubility changes or PML protein degradation [12]. In fact, transfer of PML proteins from the Radio Immunoprecipitation Assay (RIPA) lysis buffer soluble fraction to the RIPA insoluble nuclear matrix (i.e., solubility changes) by As_2_O_3_ is considered one of the most important conditions for PML protein degradation [17,18]. For instance, if following exposure to As_2_O_3_ PML protein is not shifted from RIPA soluble fraction to the RIPA insoluble matrix, it indicates that PML protein might be resistant to the As_2_O_3_ treatment.

In the current study, we have tried to find some available arsenic compounds that can degrade PML or PML mutant proteins to overcome the limitations in treatment of arsenic resistant APL patients. Here, we used phenylarsine oxide (PAO) to determine whether the mutant PML could be degraded by exposure to PAO. To prove this, three different PML expression vectors (i.e., PML-IV, PML-V and A216V) were transfected to HEK293T cells and then exposed to iAs^III^ and PAO. Although iAs^III^ is capable of inducing the normal PML-IV and -V solubility changes (i.e., induce PML protein shifted from RIPA soluble fraction to the RIPA insoluble matrix), it failed to induce the mutant PML (A216V) protein solubility changes. As anticipated, PAO has shown potential effect on induction of both normal and abnormal PML proteins solubility changes.

## 2. Results

**Figure 1:**
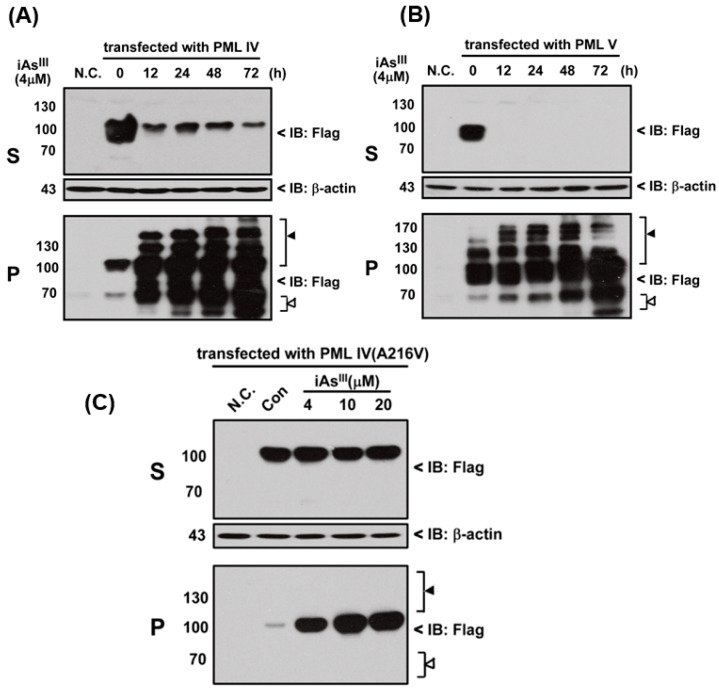
Effects of arsenite (iAs^III^) on solubility changes in PML-IV, PML-V and its mutated PML-IV (A216V) in 293T cells. HEK293T cells were transfected with plasmids encoding Flag-tagged PML-IV, PML-V and point mutated PML-IV (A216V) for 24 h. After transfection, PML-transfected cells (i.e., IV and V) were exposed to 4 µM of iAs^III^ at indicated time points (12, 24, 48 and 72 h) to determine the solubility changes of promyelocytic leukemia (PML) proteins in supernatant (S) and detergent-insoluble pellet fractions (P) by Western blot (**A**,**B**). PML-IV (A216V)-expressed cells (i.e., iAs^III^—resistant mutant PML) were treated with 4, 10 and 20 µM of iAs^III^ for 6 h, and then determine the PML protein solubility changes (**C**). The white triangle (△) indicates the protein degradation and black triangle (▲) indicates modification. PML protein and β-actin were detected by using anti-Flag and anti-β-actin primary antibodies (respectively).

In the current study, we tried to establish whether arsenite could induce the PML-IV, PML-V or mutant PML-IV (A216V) solubility changes. In this regard, three different PML plasmids were transfected into HEK293T cell and then exposed to iAs^III^ in time- and concentration-dependent manners. As expected, iAs^III^ is capable of inducing the normal PML-IV and -V proteins solubility changes, as shown in Figure 1A,B. Both PML proteins’ solubility can be shifted from RIPA soluble fraction (S) to the RIPA insoluble matrix (P) after 12-h exposure and modified or degraded in pellet fractions, suggesting that arsenic (iAs^III^) can induce the normal PML protein solubility changes (Figure 1A,B).

**Figure 2:**
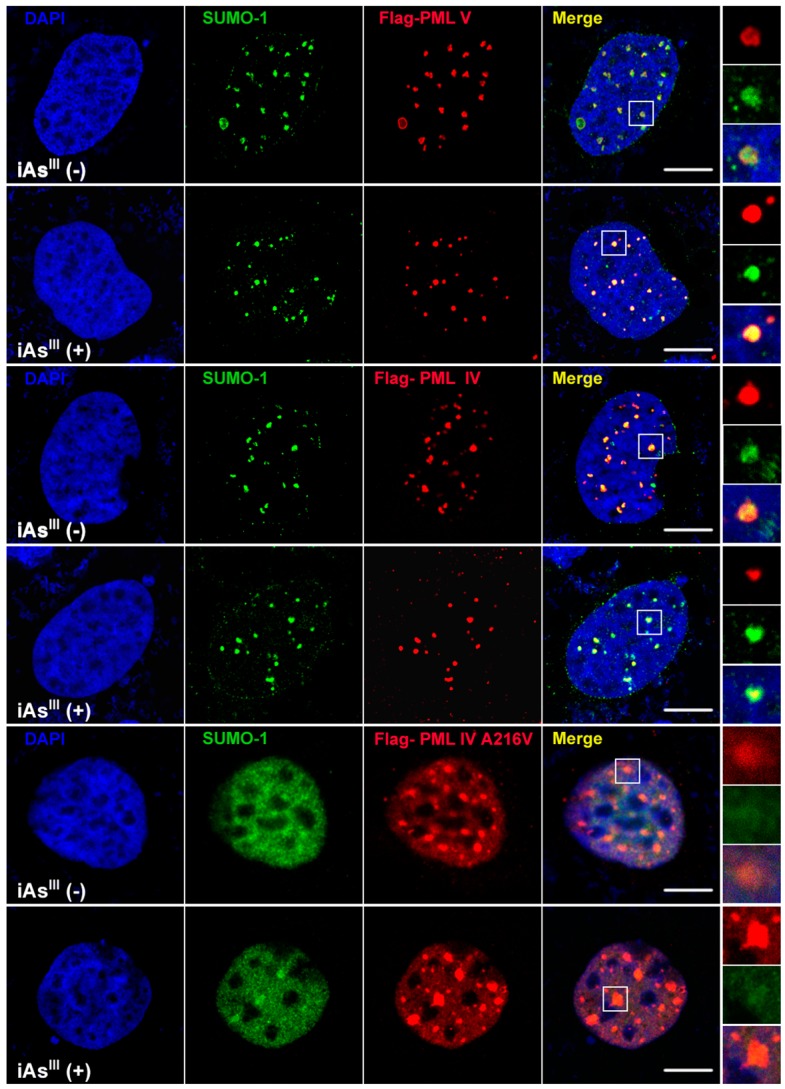
Determination of localization of PML-IV, PML-V and its mutant PML-IV (A216V) in HEK293T cells. Flag-tagged PML-IV, PML-V and point mutated PML-IV (A216V) plasmids were transfected into HEK293T cells for 24 h. After transfection, PML-transfected cells (i.e., PML-IV and -V) were exposed to 4 µM of iAs^III^ for 12 h and then determined by confocal microscopy, with PML primary antibody. Red and green fluorescence indicate PML and small ubiquitin-like modifier-1 (SUMO-1) proteins, respectively; while yellow fluorescence indicates the localization of PML and SUMO-1 proteins. Scale bar, 10 µm.

Additionally, mutant PML-IV (A216V) transfected HEK293T cells were exposed to 4, 10 and 20 µM of iAs^III^ for 6 h (Figure 1C). Our data showed that iAs^III^ cannot induce or degrade the mutated PML protein (A216V) solubility changes at high levels of arsenic (i.e., 20 µM). Likewise, formation of PML-nuclear bodies (NBs) was also determined by confocal microscopy after transfection of PML-IV, PML-V and A216V mutant in 293T cells (Figure 2). Interestingly, it was found that PML-NBs can be clearly formed by mutants PML-IV (A216V) and is similar to normal PML-IV or -V. After exposure to iAs^III^, there were no significant differences between the PML-NBs morphology changes as compared to corresponding without arsenic treatment group, indicating the PML protein (e.g., mutant) resistance to iAs^III^ is probably dependence on its protein solubility changes, but not on PML-NBs formation.

**Figure 3:**
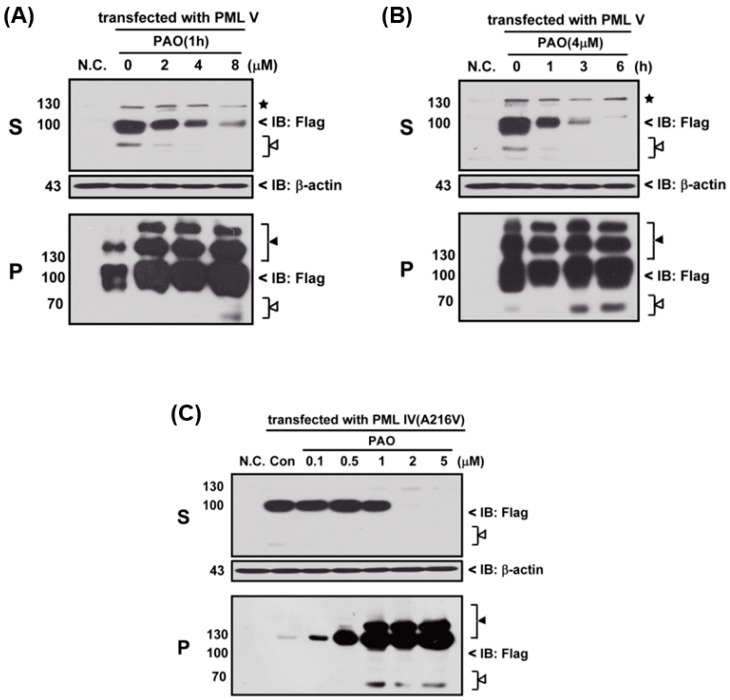
Organic arsenic compound phenylarsine oxide (PAO) is capable of inducing the arsenite-resistant mutant PML (A216V) protein solubility changes. Flag-PML-V transfected 293T cells were exposed to PAO in a concentration-dependent manner (2, 4 and 8 μM) for 1 h (**A**); or in a time-dependent manner (1, 3 and 6 h) at 4 µM (**B**) to determine the PML-V protein solubility changes. Likewise, Flag-PML-IV (A216V) transfected 293T cells were exposed to PAO at indicated concentrations (0.1, 0.5, 1, 2 and 5 μM) (**C**) to determine the protein solubility changes. The star (★) indicates non-specific protein bands, the white triangle (△) indicates the protein degradation and black triangle (▲) indicates protein modification. Protein expressions were detected using anti-Flag and anti-β-actin primary antibodies, respectively.

Next, we determined the effects of PAO on PML protein degradation, thereby three different variants of PML protein (i.e., normal and abnormal PML), PML-IV, PML-V and mutant PML-IV (A216V)-transfected 293T cells, were exposed to PAO in time- and concentration-dependent manners, as shown in Figure 3. In fact, PAO is capable of inducing PML protein solubility changes from supernatant (S) to pellet fractions (P) in both dose- and time-dependent manners (Figure 3A,B), suggesting that PAO have similar effect on normal PML degradation as like iAs^III^. However, iAs^III^ was also shown to be unable to induce the PML mutant (A216V) protein degradation [2,3]. Therefore, we attempt to determine the solubility changes of PML (A216V) protein after exposure to different concentrations of PAO, and interestingly more than 2 µM of PAO were found to be sufficient for inducing mutant PML (A216V) protein solubility changes as well as PML protein modification in pellet fractions after 6 h exposure (Figure 3C), indicating that PAO have strong dual effects on both normal or abnormal PML protein solubility changes along with PML proteins degradation.

**Figure 4:**
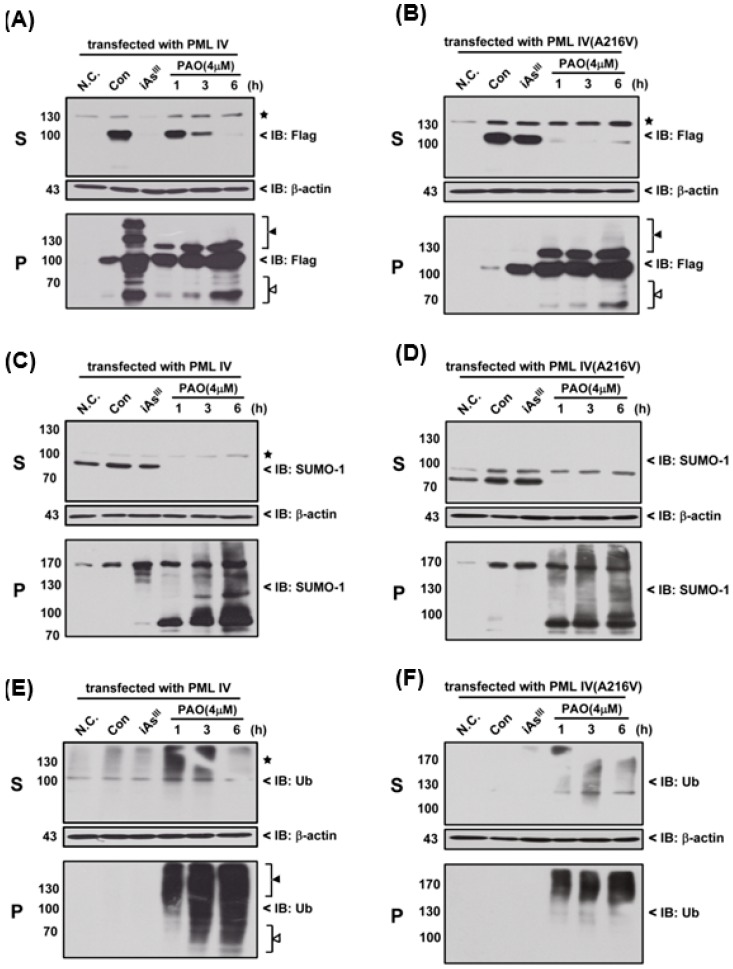
Effects of PAO on induction of SUMOylation and ubiquitination. Flag-PML-IV or PML-IV (A216V) over-expressed HEK293T cells were treated with 4 µM iAs^III^ for 6 h or PAO at indicated time points (1, 3 and 6 h) to determine the PML-IV (**A**); and PML-IV (A216V) (**B**) protein solubility changes; SUMOylation (**C**,**D**); and ubiquitination (**E**,**F**). The star (★) indicates non-specific protein bands, the white triangle (△) indicates the protein degradation and black triangle (▲) indicates protein modification. SUMOylation and ubiquitination of PML protein in supernatant and pellet fractions were detected using anti-SUMO-1 and anti-Ub primary antibodies, respectively.

Moreover, we used 4 µM of PAO to compare the differences (e.g., SUMOylation and ubiquitination) between the normal PML and mutant PML (A216V) at different time periods, as shown in Figure 4. Interestingly, it seems that mutant PML protein was more sensitive to PAO treatment than normal PML-IV at 1 h exposure (Figure 4A,B).

On the other hand, PAO (but not of iAs^III^) has completely induced SUMO-1 protein solubility changes from S to P fractions in two different PML transfected cells (Figure 4 C,D). Likewise, PAO also dramatically induced PML protein ubiquitination in supernatant and pellet fractions when compared to iAs^III^ (Figure 4E,F), indicating that induction of PML protein SUMOylation and ubiquitination by PAO are much stronger than iAs^III^.

**Figure 5:**
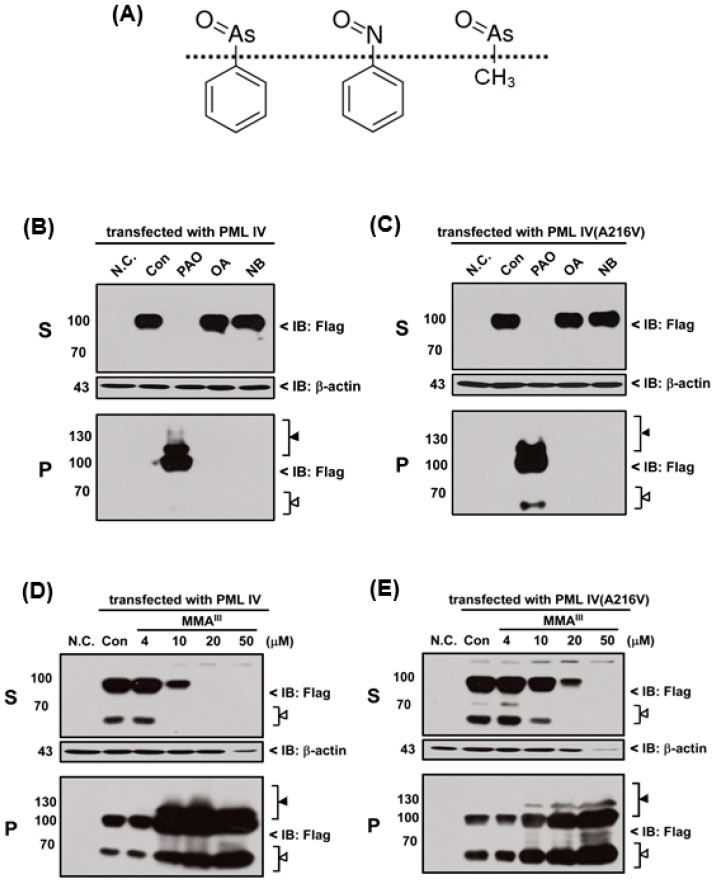
Comparison of different chemical compounds on induction of PML-IV or mutant PML-IV (A216V) proteins solubility changes. (**A**) The molecular structures of PAO, Nitrosobenzene (NB) and monomethylarsonous acid (MMA^III^). Flag-PML-IV or PML-IV (A216V) over-expressed HEK293T cells were treated with 4 µM phenylarsine oxide (PAO), 100 nM Okadaic acid (OA) and 4 μM Nitrosobenzene (NB) for 6 h; and then the PML-IV (**B**); and PML-IV (A216V) (**C**) protein solubility changes were determined by Western blot. Similarly, PML-IV (**D**); and PML-IV (A216V) (**E**) protein solubility changes were also determined in case of MMA^III^ treatment in a concentration-dependent manner (i.e., 4, 10, 20 and 50 μM). The white triangle (△) indicates the protein degradation and black triangle (▲) indicates protein modification. PML protein and β-actin were detected using anti-Flag and anti-β-actin primary antibodies, respectively.

**Figure 6:**
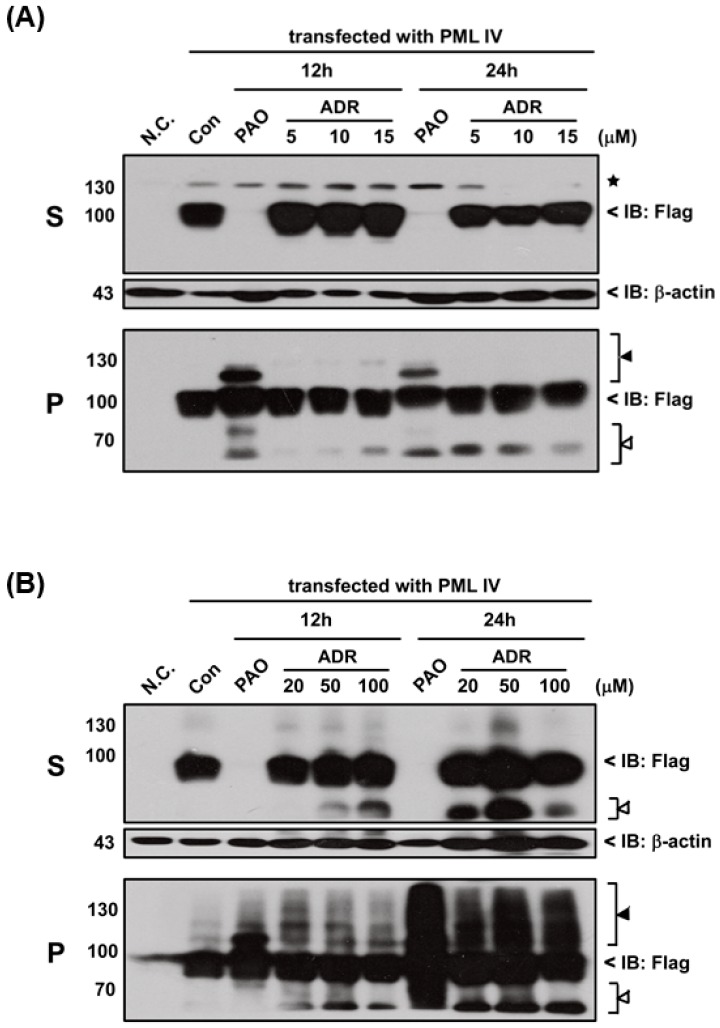
Effect of adriamycin (ADR) on induction of PML-IV protein solubility changes in 293T cells. Flag-PML-IV overexpressed 293T cells were exposed to: (**A**) low (i.e., 5, 10 and 15 µM); and (**B**) high concentrations (i.e., 20, 50 and 100 µM) of ADR for 12 or 24 h. Additionally, in both cases, 4 μM of PAO were also used to compare the effects of PAO with adriamycin on PML-IV protein solubility changes. After treatment, PML-IV protein solubility changes were determined by Western blot with Flag antibody. The star (★) indicates non-specific protein bands, the white triangle (△) indicates the protein degradation and black triangle (▲) indicates protein modification.

In fact, PAO is a protein phosphatase inhibitor [24] and contains a phenyl group. Thus, we hypothesized that inhibition of protein phosphatase activity or phenyl group of PAO may be involved in PML protein solubility changes. Therefore, three different chemical compounds such as phenylarsine oxide (PAO), okadaic acid (OA; inhibitor of protein phosphatase) and nitrosobenzene (NB; contain phenyl group) were used to compare the PML-IV or mutant PML (A216V) protein solubility change, as shown in Fgiure 5A. Unexpectedly, OA and NB have no considerable effects on the induction of either PML protein solubility changes when compared to PAO (Figure 5B,C). Surprisingly, it was found that trivalent methylated MMA^III^ at high dose (i.e., more than 20 µM) is capable of inducing both PML proteins solubility changes (Figure 5D,E), because there are structural similarities between MMA^III^ and PAO. To understand whether PAO and MMA^III^ induced PML protein solubility changes are specific, normal and abnormal *PML* genes transfected cells were exposed to DMA^III^ or adriamycin (ADR; a chemotherapeutic agent). However, PML proteins solubility changes were not found after exposure to either DMA^III^ (Figure S1) or ADR (Figure 6) even though they cause cell death (Figure S2), indicating that the PAO induced PML protein solubility changes are different from other chemical compounds.

## 3. Discussion

PML or PML-RARα protein solubility changes, hyper-SUMOylation and degradation in response to As_2_O_3_ (i.e., iAs^III^) constitute the molecular mechanisms of arsenic-induced treatment of APL [14,25]. Unfortunately, some patients still develop arsenic resistance during their therapy and are often found in relapsed APL. Moreover, their outcomes are also extremely poor. Recent studies have clearly revealed that genetic mutations in B-box2 moiety of *PML* gene rendered resistance to As_2_O_3_ [26]. Thus, new arsenic compounds are needed to investigate which will be more effective than As_2_O_3_, and can induce the mutated PML protein solubility changes and degradation.

In fact, PML protein solubility changes by As_2_O_3_ are suggested to be the most important and critical step for PML protein degradation in response to As_2_O_3_ treatment [11]. For instance, if the mutant PML protein is resistant to As_2_O_3_ in vitro (i.e., no solubility changes), their corresponding PML mutants in APL patients also exhibit resistance to As_2_O_3_ [12,17]. In this study, we found that iAs^III^ can induce normal PML-IV and -V solubility changes, and PML-IV seems to be less sensitive to iAs^III^ treatment than PML-V (Figure 1). Additionally, iAs^III^ had no appreciable effects on induction of solubility changes of mutant PML (A216V), which is consistent with other published reports [16,17,18]. On the other hand, mutant PML (A216V) protein can form PML-NBs but it cannot be degraded by iAs^III^, suggesting that formation of the PML-NBs is not a sufficient condition for mutant PML protein degradation (Figure 2).

Phenylarsine oxide (PAO) is a membrane-permeable protein-tyrosine phosphatase (PTPase) inhibitor [24]. An report has indicated that PAO is capable of inhibiting proliferation or inducing apoptosis of AML cells in vitro [27]. This efficacy of PAO against AML cells increased interest in using it for the treatment of As_2_O_3_ resistant APL. Therefore, we attempted to examine whether PAO could induce mutant PML protein solubility changes; thereby, normal and abnormal PML overexpressed 293T cells were exposed to PAO in dose- and time-dependent manners (Figure 3). Interestingly, we found that PAO can induce both normal and abnormal PML proteins solubility changes, especially PAO significantly increased PML protein modification in pellet fractions. Notably, low dose of PAO (i.e., 0.5–1 µM) had no appreciable effect on induction of mutant PML (A216V) protein solubility changes, while 2 µM of PAO were found to be sufficient to induce mutant PML protein solubility changes (S to P), indicating that PAO may have good effects on mutant PML protein degradation. Particularly, we also observed that mutant PML (A216V) protein is more sensitive to PAO than normal PML (Figure 3).

PML or PML-RARα protein degradation by As_2_O_3_ was identified to be mainly through SUMOylation and ubiquitination dependent proteasome pathways [13,14]. Additionally, small ubiquitin-related modifier-1 (SUMO-1) is a well-characterized member of the family of ubiquitin-related proteins, and PML protein is modified by SUMOylation at three important lysine residues (K65, K160 and K490 present in corresponding regions as RING, B-box 1 and carboxy-terminal region) after exposure to As_2_O_3_ [28]. In the current study, we found that PAO can dramatically induced PML protein SUMOylation and ubiquitination (Figure 4), suggesting that normal or mutant PML might be degraded through SUMOylation and ubiquitination proteasome pathways after PML protein solubility changes.

Moreover, we hypothesized that inhibition of protein phosphatase activity or phenyl group of PAO may be involved in PML protein solubility changes, therefore inhibitor of protein phosphatase OA and nitrosobenzene (NB) were used to examine the normal and mutant PML protein solubility changes. However, neither NB nor OA could induce the PML protein solubility changes (Figure 5). On the other hand, in our previous study, we reported that low dose exposure of methylated arsenicals (i.e., MMA^III^ and DMA^III^) has no appreciable effect on the PML or PML-RARα fusion protein solubility changes [29]. In this study, high concentrations of iAs^III^, MMA^III^ and DMA^III^ were used to establish whether these arsenicals could induce the mutant PML protein degradation similarly to PAO. Unexpectedly, 50 µM of iAs^III^ or 50 µM of DMA^III^ failed to induce the mutant PML protein solubility changes (Figure S1). Surprisingly, high concentrations of MMA^III^ induced PML proteins solubility changes similarly to PAO (Figure 5).

Here, in order to confirm where PAO (or MMA^III^) induced PML protein solubility changes are dependent on cell death by cytotoxicity, high concentrations (e.g., 100 µM) of adriamycin, a chemotherapeutic agent, was used to establish PML protein solubility changes (Figure 6). Interestingly, there is no PML protein solubility changes observed by exposure to adriamycin even though it showed high toxicity (Figure S2), suggesting that PAO induced PML protein solubility changes may depend on another mechanism.

## 4. Materials and Methods

### 4.1. Reagents

All reagents were of analytical grade. Milli-Q water (Millipore, Bedford, MA, USA) was used throughout the experiment. Trizma^®^ HCl and Trizma^®^ Base, phenazene methosulfate, decylubiquinone, 3-(4,5-dimethylthiazol-2-yl)-2,5-diphenyl tetrazolium bromide (MTT), and phenylarsine oxide (PAO) were purchased from Sigma (St. Louis, MO, USA). Sodium arsenite (iAs^III^), dimethylarsinic acid [(CH_3_)_2_AsO(OH)] (DMA^V^) were purchased from Wako Pure Chemical Industries, Ltd. (Osaka, Japan). Monomethylarsonic acid (MMA^V^) was obtained from Tri Chemicals (Yamanashi, Japan). The arsenic standard solutions were stored in the dark at 4 °C. Diluted standard solutions for experiments were prepared daily prior to use.

### 4.2. Preparation of Trivalent Monomethylarsonous Acid (MMA^III^) and Dimethylarsinous Acid (DMA^III^)

MMA^III^ and DMA^III^ were prepared by reducing MMA^V^ and DMA^V^, respectively, with 5 molar equivalents of l-cysteine in distilled water at 90 °C for 1 h. The trivalent forms were confirmed by comparison of the respective retention times on a GS 220 gel filtration column by HPLC-ICP MS (Showa Denko, Tokyo),with those prepared from their iodide forms in distilled water under nitrogen atmosphere. Purity of MMA^III^ (98%, with 2% of MMA^V^) and DMA^III^ (95%, and with 5% of DMA^V^) was confirmed by HPLC-ICP MS, and then used.

### 4.3. Confirmation of Arsenic Species by HPLC-ICP MS

The HPLC system consisted of a liquid chromatograph solvent delivery PU-610 pump and a DG 660B-2 degasser (GL Sciences Co., Tokyo, Japan). A strong anion exchange column (Hamilton PRPX-100, 5 μm 4.1 mm × 150 mm) was used to detect the arsenic species. A 20-µL aliquot of a sample solution was applied to the PRPX-100 column, and then the column was eluted with a mobile phase of 35 mM ammonium bicarbonate, pH adjusted to 8.2, with a flow rate of 0.8 mL/min (PRP X-100). Arsenic in the eluate was monitored with an Agilent HP7500cs ICP MS (Yokogawa, Hachiouji, Japan) equipped with an octopole reaction system (ORS) with a He flow of 3.0 mL per min to prevent molecular interference by ^40^Ar^35^Cl (signal at *m*/*z* 75).

### 4.4. Cell Culture

HEK293T cells were purchased from Cell Bank of China Science. Following receipt, cells were grown and frozen as a seed stock as they were available. Cells were passage for a maximum of 3 months, after which new seed stocks were thawed. Two cell lines were authenticated using DNA fingerprinting (variable number of tandem repeats), confirming that no cross-contamination occurred during this study. Two cell lines were tested for mycoplasma contamination at least two times per year.

Cells (1.0 × 10^6^) were cultured in T25 flask, and were maintained in logarithmic growth phase using RPMI-1640 or Dulbecco’s Modified Eagle Medium (DMEM) supplemented with 10% fetal bovine serum (FBS), 100 U/mL penicillin, and 100 µg/mL streptomycin, at 37 °C in 5% CO_2_ atmosphere. After 24 h of seeding, cultures were washed with PBS, fresh medium was added, and the cells were treated with indicated doses of arsenicals and all-trans retinoic acid for indicated time.

### 4.5. Western Blot Analysis

HEK 293T cells were lysed in RIPA buffer without 8 mole (M) urea, and then incubated on ice and centrifuged as mentioned above to obtain the supernatant and pellet fractions. The supernatant was transferred and the pellet was washed with PBS, lysed in SDS buffer [1× tris-buffered saline ((TBS), 10% glycerol, 0.015% ethylenediaminetetraacetic acid (EDTA), 50 mM dithiothreitol (DTT), and 2% sodium dodecyl sulfate (SDS)], boiled for 10 min at 95 °C. Twenty-five microgram of each protein sample was resolved by 10%–12% SDS-PAGE and electroblotted onto nitrocellulose membranes. The membranes were blocked with non-fat milk and incubated overnight with different primary antibodies at 4 °C, followed by incubation with horseradish peroxidase (HRP)-linked secondary antibodies for 1 h at room temperature and then the proteins were visualized by enhanced chemiluminescence.

### 4.6. Immunofluorescence Microscopy

*FLAG-PML* transfected HEK293T cells were cultured on Chamber slides and then exposed to arsenicals. After washing with phosphate-buffered saline (PBS) twice, the slides were fixed in 4% paraformaldehyde and permeablized with 0.1% Triton X-100, blocked with PBST with 10% fetal bovine serum. Further, the cells were then blocked with 1% bovine serum albumin (BSA) in PBS, followed by incubation with primary antibodies overnight at 4 °C. Later, they were tagged with fluorescent secondary antibodies for 2 h. Slides were mounted using Vectashield mounting liquid (Vector Labs, Burlingame, CA, USA), sealed, and stored in dark at 4 °C. The cells were examined under a Zeiss (Göttingen, Germany) 510 confocal microscope.

## 5. Conclusions

In the current study, we, for the first time, found that PAO is capable of inducing mutant PML protein solubility changes and degradation, suggesting that PAO is a considerable agent for relapsed/refractory APL. However, further investigation is needed to determine the dose level and toxicity of PAO in vivo and in vitro more carefully for clinical practices.

## Supplementary Materials

Supplementary materials can be found at www.mdpi.com/1422-0067/18/2/247/s1.

## Abbreviations

iAs^V^	arsenate
iAs^III^	arsenite
APL	acute promyelocytic leukemia
DMA^III^	dimethylarsinous acid
DMA^V^	dimethylarsinic acid
MMA^III^	monomethylarsonous acid
PAO	phenylarsine oxide
