# Supplementary material for: Phenylarsine Oxide Can Induce the Arsenite-Resistance Mutant PML Protein Solubility Changes
Source: Int J Mol Sci. 2017 Jan 25;18(2):247. doi: 10.3390/ijms18020247 (PMC5343784; doi:10.3390/ijms18020247)
Supplement: Supplementary file 1 [file ijms-18-00247-s001.pdf]

Supplementary Material: Phenylarsine Oxide Can Induce The Arsenite-Resistance Mutant PML Protein Solubility Changes

Yu Han Jiang, Ye Jia Chen, Chao Wang, Yong Fei Lan, Chang Yang, Qian Qian Wang, Liaqat Hussain, Yasen Maimaitiying, Khairul Islam and Hua Naranmandura

Cytotoxicity Assay: Trypan Blue Dye-Exclusion Test

HEK 293T cells (state of confluence: max. 90%) were treated with different concentrations of adriamycin and DMA^{III} for 12 h. Cell viability was evaluated after exposure. Treated and untreated cells were harvested by trypsin treatment. Cell counting was performed following Trypan blue staining. The cell suspension was mixed with a 1/4th volume of 0.4% Trypan blue solution and subsequently evaluated under the light microscope. The membrane of dead cells is permeable to Trypan blue (blue stained cells), whereas living cells remain unstained. Cell viability is expressed as percentage of surviving cells compared to the total number of cells.

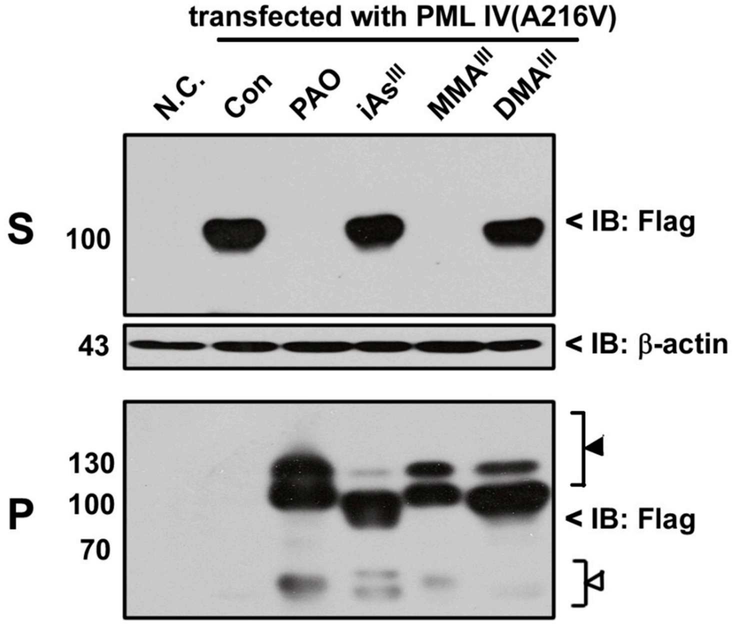

Figure S1. Flag-PML-IV (A216V) overexpressed 293T cells were exposed to 4 μM PAO or each 50 μM of iAs^{III}, MMA^{III} and DMA^{III} for 12 h. The changes of protein expressions in soluble and insoluble fractions were detected by Western blot with Flag antibody. The white (△) and black (▲) triangles indicate protein degradation and modification, respectively.

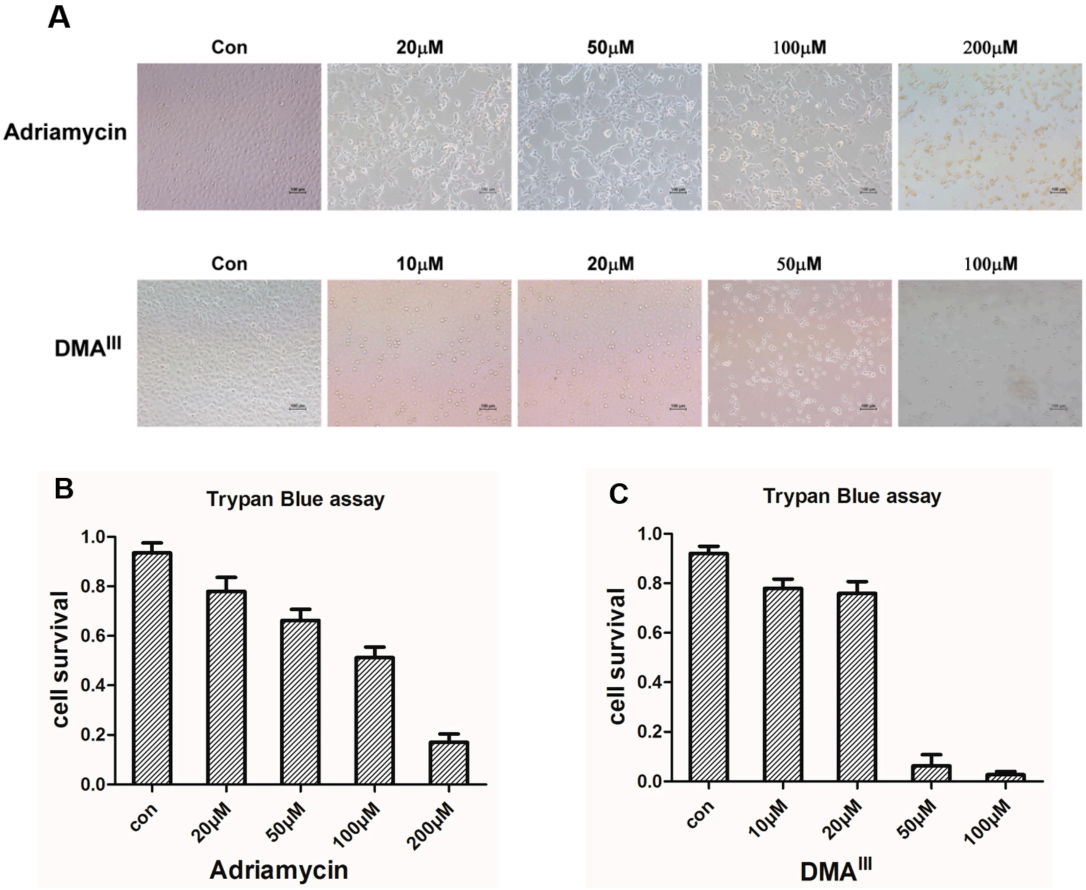

Figure S2. Morphological changes of 293T cells after treatment with different concentrations of adriamycin and DMA^{III} for 12 h (**A**). Cell survival was determined by Trypan Blue staining assay after: Adriamycin exposure (**B**); and DMA^{III} exposure (**C**). Scale bar, 100 μm.
